# Letrozole and Crocin: Protecting Leydig Cells and Modulating Androgen Receptor and CYP19 Gene Expression in Busulfan-Induced Azoospermia

**DOI:** 10.3390/ani15050697

**Published:** 2025-02-27

**Authors:** Shahrzad Nokhbeh Zaeem, Mitra Heydari Nasrabadi, Masoud Salehipour, Somayeh Ehtesham

**Affiliations:** Department of Biology, Faculty of Biological Sciences, Parand Islamic Azad University, Parand 3761396361, Iran; shahrzad.nz1992@gmail.com (S.N.Z.); m.salehipour@piau.ac.ir (M.S.); somayehtesham@gmail.com (S.E.)

**Keywords:** azoospermia, antioxidants, busulfan, crocin, letrozole, synergistic, CYP19

## Abstract

This study aimed to investigate the impact of letrozole and crocin on Leydig cells on busulfan-induced azoospermia using a rat model. A sample population consisting of thirty male rats was randomly assigned to five groups: (1) the control group, (2) azoospermia group, (3) azoospermia group treated with letrozole, (4) azoospermia group treated with crocin, and (5) azoospermia group treated with both letrozole and crocin. The treatment period with letrozole and crocin lasted for four weeks following busulfan administration. Subsequently, comprehensive analyses, including histopathological, molecular, and hormonal assessments, were performed, followed by immunohistochemical staining. This study found that the control group exhibited normal Leydig cell morphology, while the azoospermia group showed reduced Leydig cells and tissue disruptions. Letrozole and crocin treatments were associated with increased testicular fibrosis in the AZO and AZO + Cro groups, while their combination notably reduced fibrosis to approximately 15%. Furthermore, the combination treatment enhanced antioxidant enzyme activity and upregulated androgen receptor expression. Although a number of improvements were noted in sperm motility, they were not statistically significant. Further research is required to clarify the therapeutic implications of these findings in azoospermia treatment.

## Simple Summary

This study explored the impact of letrozole and crocin on Leydig cells in male rats with azoospermia induced by busulfan, a chemotherapy drug used to treat certain cancers. Azoospermia is a condition where sperm is absent in semen and is a major cause of male infertility. Researchers used rats during the experiments, dividing them into different groups. The rats received letrozole, crocin, or both after exposure to busulfan. The study found that while letrozole and crocin did not significantly restore cell viability, they improved sperm motility. The combination treatment also enhanced antioxidant activity, which protects against oxidative stress. Additionally, the treatment increased androgen receptor expression and CYP19A gene activity, both important for hormone balance and sperm production. In contrast, the busulfan-treated rats showed significant declines in these markers, indicating damage to testicular function. The findings suggest that letrozole and crocin may work together to support recovery from azoospermia. However, further research is needed to fully understand their effectiveness and potential for clinical use in male infertility treatment.

## 1. Introduction

Male infertility is associated with various symptoms, including reduced sperm production, impaired sperm function, and obstruction in transport pathways [1]. Non-obstructive azoospermia (NOA) is among the key causes of male infertility. NOA is defined as the absence of sperm in ejaculate [2], accounting for 10 to 15% of male infertility cases. Approximately 75% of men with infertility experience issues related to sperm production, many of which are regrettably untreatable [3]. However, despite severe disturbances in spermatogenesis and the absence of sperm in ejaculation, focal areas of spermatogenesis can be identified in 30 to 60% of affected men. In such cases, the possibility of natural fertilization and embryo development exists following sperm retrieval [4].

The clinical and social treatment of NOA-affected men, particularly those who lack sperm count in their test results and desire fatherhood, remains a challenging issue and is further complicated since NOA can stem from a variety of causes, ranging from genetic disorders to hormonal imbalances [5]. The administration of busulfan medication is one such cause with a significant impact on male fertility, exhibiting varying symptoms depending on the administered dosage [6]. A number of studies have documented the presence of identifiable sperm in patients treated with busulfan following bone marrow transplantation, while other researchers have reported oligospermia and low testosterone levels [7]. Conversely, studies indicate that low doses of busulfan, administered following hematopoietic stem cell transplantation, have a minimal impact on male reproductive functions, with only a small percentage of patients experiencing reduced testosterone levels [8]. Findings from animal studies corroborate these results, demonstrating that low doses of busulfan effectively induce azoospermia in mice. This evidence suggests that the loss and recovery of fertility associated with busulfan administration is directly linked to the reduction and regeneration of spermatogonial stem cells (SSCs) [9]. Thus, understanding the threshold for SSC recovery could be critical in developing clinical strategies aimed at preserving post-chemotherapy male fertility [9].

Moreover, certain studies have demonstrated that busulfan disrupts the redox balance and triggers oxidative damage, although antioxidants may mitigate these effects [10]. The deficiencies in previous studies may contribute to the inadequate success of their proposed azoospermia treatment methods. These deficiencies include failure to account for the plasticity and recovery of spermatogonial stem cells, as well as appropriate combinatory therapies for enhancing fertility [11].

Aromatase inhibitors, such as letrozole, are utilized to prevent androgen-to-estrogen conversion, thereby increasing testosterone levels and potentially improving male infertility [12]. The choice to combine letrozole and crocin is driven by their complementary benefits. Specifically, letrozole enhances the spermatogenic processes by lowering estrogen levels and raising testosterone [13]. Moreover, given its antioxidant properties, crocin can prevent oxidative damage and maintain the health of Leydig cells [14].

Saffron (*Crocus sativus*) is rich in antioxidant compounds like crocin and offers several therapeutic benefits, including enhanced sexual performance and protection against oxidative stress-induced cell death [15]. Oxidative stress, induced by factors such as busulfan, adversely impacts sperm quality through mechanisms such as causing DNA damage and disrupting spermatogenesis [16]. Ongoing studies into the hormonal impacts on sperm production have led to the development of novel therapeutic strategies for male infertility involving various hormonal medications [17]. Among these, letrozole shows promise in idiopathic male infertility treatment, presenting a compelling area of study.

The present study proposes significant innovations compared to previous research [18]. First, it investigates the combined impact of letrozole and crocin on Leydig cells in a busulfan-induced azoospermia model, whereas numerous earlier studies have focused on these compounds individually. Second, the study provides deeper insights into the molecular mechanisms involved, particularly alterations in androgen receptor expression and the *CYP19A1* gene, enhancing our understanding regarding the impact of said compounds on male fertility. The findings of this study can contribute to the development of novel and improved azoospermia treatments, thus improving patient quality of life, a critical aspect of infertility treatment. Consequently, this study aims to investigate the protective impact of letrozole and crocin on Leydig cells in the busulfan-induced azoospermia model while examining the changes in *androgen receptor* expression and *CYP19A1* gene activity.

## 2. Materials and Methods

The laboratory assays employed a range of materials and equipment across several techniques. For Real-Time PCR, key components included SYBR TM (2X) Master Mix (Thermo Fisher Scientific, Waltham, MA, USA), forward and reverse primers (Integrated DNA Technologies, Coralville, IA, USA), and cDNA synthesis reagents (Invitrogen, Carlsbad, CA, USA). The TAC assay utilized RIPA buffer (Cell Signaling Technology, Danvers, MA, USA) and standard solutions (Merck, Darmstadt, Germany). The TOS assay included TOS Buffer (Abcam, Cambridge, UK) and Developer Solution (Cayman Chemical, Ann Arbor, MI, USA). For RNA extraction, RiboEx solution (GeneAll Biotechnology, Seoul, South Korea) and buffers from Qiagen (Hilden, Germany) were used. Additional reagents included those for Masson’s trichrome staining and sperm assessments. Equipment included centrifuges and microtubes (Eppendorf, Hamburg, Germany), light microscopes (Olympus, Tokyo, Japan), an autoclave (Getinge, Gothenburg, Sweden), and an incubator (Thermo Fisher Scientific, Waltham, MA, USA).

### 2.1. Animal Preparation

Thirty adult male Sprague Dawley rats (8–10 weeks old, 180–220 g; source: Pasteur Institute of Tehran, Tehran, Iran) were utilized in this study. The rats were housed under a controlled environment with monitored temperature and humidity set to 25 °C and 40–50%, respectively. Food and water were provided ad libitum.

### 2.2. Crocin Preparation

Crocin (Sigma-Aldrich, St. Louis, MO, USA).was dissolved in distilled water to reach a final concentration of 10 mg/mL. The dosage was calculated based on rat weight and administered intraperitoneally [19]. In this study, administering 10 mg of crocin to a rat weighing 0.2 kg results in a dosage of 50 mg/kg, calculated using the following formula: dose per kilogram = amount of drug (mg)/weight (kg).

### 2.3. Experimental Design

Rats were divided into five groups (n = 6 per group): (1) control group, (2) azoospermia group (busulfan: Sigma-Aldrich: St. Louis, MO, USA), (3) azoospermia group treated with letrozole (Novartis: Basel, Switzerland) (1 mg/kg body weight for 3–4 weeks), (4) azoospermia group treated with crocin, and (5) azoospermia group treated with both letrozole and crocin. Male desert rats were subjected to daily intraperitoneal injections of 10 mg of busulfan for 10 days to model spermatogenic impairment, which is characterized by reduced sperm production [20]. While the treatment induced significant changes in sperm production, sperm viability was still evaluated throughout the study. Letrozole was administered from day 11 to day 31, while crosin was concurrently prescribed during the final week of letrozole treatment. At the conclusion of the treatment period, the animals were sacrificed for subsequent analysis of sperm viability and other parameters.

### 2.4. Hematoxylin–Eosin and Masson’s Trichrome Staining

Testis samples were decalcified in 10% EDTA solution, dehydrated through graded alcohol series, and embedded in paraffin wax. Sections with 5 µm thickness were stained with hematoxylin–eosin and Masson’s trichrome.

### 2.5. Sperm Functional Parameter Assessment

Sperm viability was assessed using the hypo-osmotic swelling test (HOST). The motility and viability of mature sperm samples were evaluated under a light microscope.

### 2.6. Immunohistochemistry (IHC) Analysis

Blood samples were collected to determine the total antioxidant capacity (TAC) using the ferric reducing antioxidant power (FRAP) assay. Samples were then centrifuged using the TAC Assay Kit (Abcam: Cambridge, UK).

### 2.7. Measurement of Testosterone Levels

Testosterone levels in serum samples were measured using a Free Testosterone kit. Absorbance was measured at 450 nm following incubation with testosterone and anti-testosterone solutions.

### 2.8. Gene Expression Assessment Using Real-Time Quantitative PCR (qPCR)

Total RNA was extracted from the testis samples using a standardized protocol. Subsequently, complementary DNA (cDNA) was synthesized utilizing a commercially available cDNA Synthesis Kit. Real-time quantitative PCR (qPCR) was carried out to quantify the expression levels of the androgen receptor (r-androgen receptor) and cytochrome P450 aromatase (r-CYP19A) genes. Data analysis was performed using the OligoV7.60 software (https://www.oligo.net), facilitating the evaluation of gene expression relative to appropriate housekeeping genes (Table 1).

### 2.9. Statistical Analysis

Data are presented as mean ± standard deviation. One-way analysis of variance (ANOVA) followed by Tukey’s post hoc test was used to compare means (*p* < 0.05).

## 3. Results and Discussion

**Figure 1:**
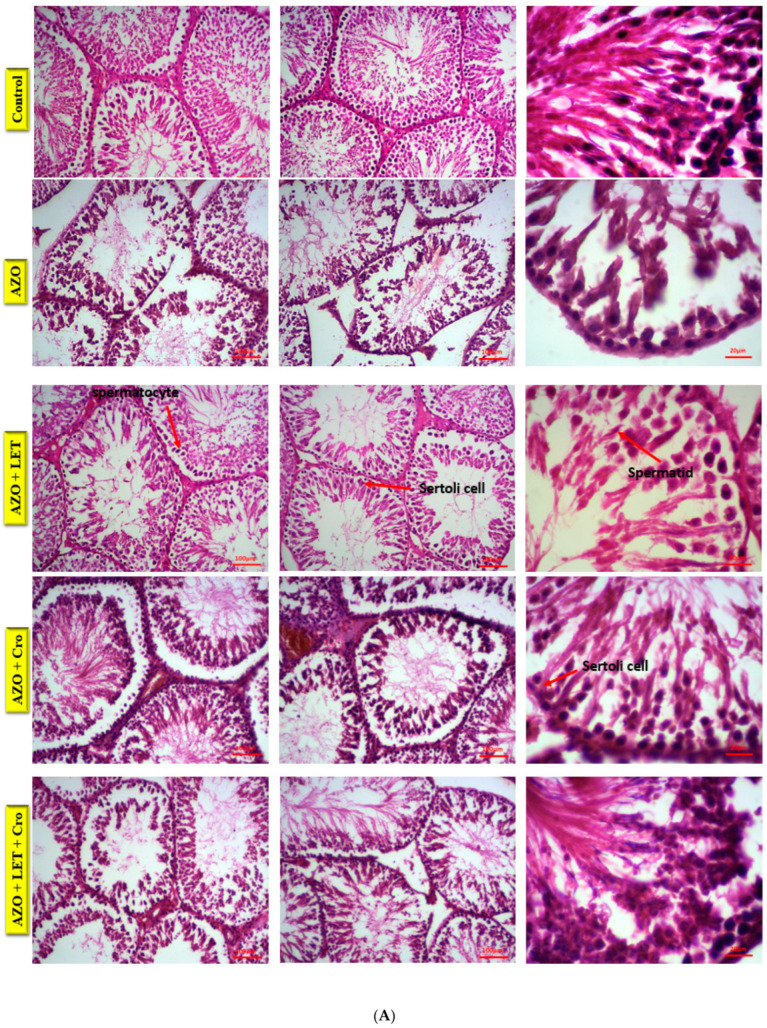

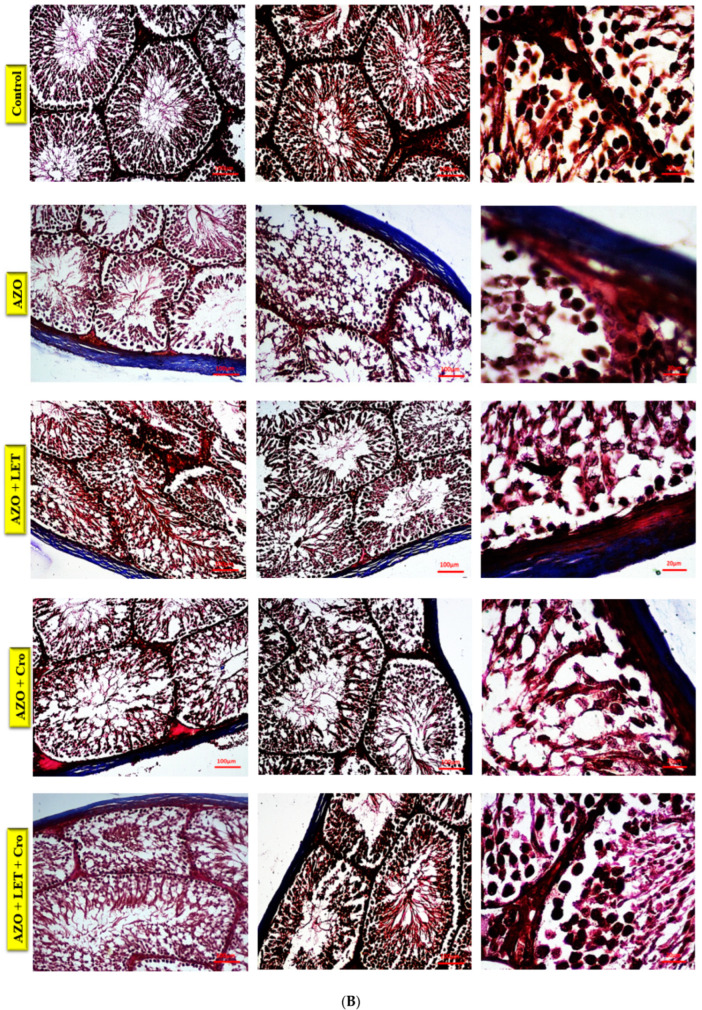
Microscopic images of testicular tissue from male rats in different treatment groups were analyzed using 5 µm sections stained with hematoxylin–eosin (H&E) at ×1000 magnification (**A**) and Masson’s trichrome stain (**B**). Control group exhibited normal testicular morphology. “Azo” group (busulfan 10 mg/kg) showed significant histopathological alterations. “Azo + Let”, “Azo + Cro”, and “Azo + Let + Cro” groups displayed combined effects of busulfan with letrozole, crocin, and both drugs, respectively. Masson’s trichrome staining further visualized testicular capsule.

The control rat sample exhibited normal Leydig cell morphology and functionality (Figure 1A), essential for spermatogenesis and testosterone production. In contrast, the azoospermia group exhibited significant Leydig cell loss, cytoplasmic degeneration, and basement membrane damage, disrupting the testicular microenvironment and germ cell integrity (Figure 1A).

Letrozole and crocin treatments (Figure 1A) mitigated these effects, preserving Leydig and germ cells.

In this study, Masson’s trichrome staining was employed to distinguish collagen fibers from other tissue components, namely cytoplasm-stained red and nuclei-stained black. Additionally, the degree of fibrosis, characterized by the excessive accumulation of collagen and other extracellular matrix proteins, was assessed. The ANOVA confirmed the statistical significance of these differences (*p* < 0.0001). The post hoc analysis (Tukey’s test) demonstrated a significant difference concerning fibrosis compared to both the control and the crocin-only group. However, this difference does not significantly vary from the letrozole-only group, suggesting that letrozole is the primary driver of the observed fibrotic response (Figure 1B)

**Figure 2:**
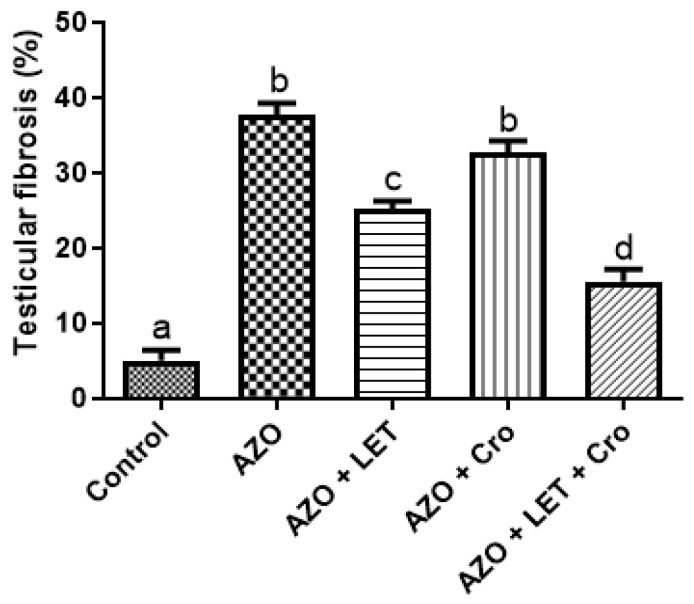
The results of Masson’s trichrome staining, along with the observed changes in the rat testicular capsules in the study group, indicate that groups with similar lettering do not show significant differences. The letters (a, b, c, etc.) above the bars in the graph represent statistically significant groupings. Bars with identical letters are not significantly different, while bars with dissimilar letters have a statistically significant difference (at the *p* < 0.05 level, as typically indicated in the figure legend or Materials and Methods Section).

The bar graph illustrates that the azoospermia (AZO) group exhibited significantly higher testicular fibrosis (approximately 37%) compared to the control group (approximately 5%). Letrozole (LET) treatment alone reduced fibrosis to around 26%, while administering crocin (Cro) alone resulted in fibrotic levels of approximately 34%. Notably, the combined treatment of letrozole and crocin (AZO + LET + Cro) led to a substantial reduction in fibrosis, reaching levels of around 15% (Figure 2).

These findings align with previous studies highlighting the protective impact of letrozole and crocin on Leydig cell populations in oxidative stress-induced azoospermia, enhancing testosterone synthesis and spermatogenesis [21]. Additionally, the combination of letrozole and crocin mitigates the cytotoxic impact of chemotherapeutic agents like busulfan, thus preserving testicular architecture and improving germ cell layer thickness [22]. The integrity of Leydig cells is crucial for testosterone production and overall male reproductive health [23]. Furthermore, fibrotic changes observed in the azoospermia + letrozole group indicate disruptions in hormonal signaling and spermatogenesis. In contrast, enhanced vascular remodeling in the combination group supports tissue repair and functionality [24]. Overall, these findings highlight the therapeutic potential of letrozole and crocin in azoospermia treatment, underscoring the significance of multifaceted therapeutic approaches for male infertility treatment [25].

**Figure 3:**
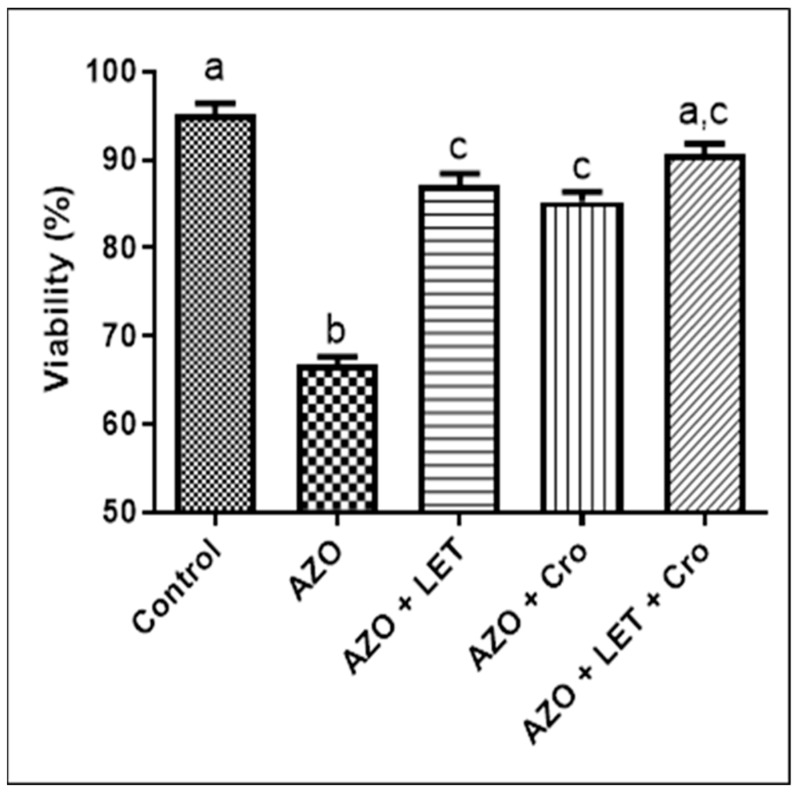
Sperm viability assessment results revealed significant changes. Dissimilar letters denote statistical differences (*p* < 0.05). Letters (a, b, c, etc.) above bars represent statistically significant groupings. Bars with identical letters are not significantly different, while bars with dissimilar letters have statistically significant difference (at *p* < 0.05 level, as typically indicated in figure legend).

This study presents a comprehensive analysis of the impact of azoospermia on cell viability and sperm motility, highlighting the complexities of male infertility. Azoospermia is characterized by the absence of sperm in the ejaculate and poses significant challenges to male reproductive capabilities, underscoring the need for a deeper understanding of its mechanisms and potential treatments. This study revealed a substantial decline in cell viability in azoospermia conditions compared to the control group, highlighting the urgent need for effective treatment strategies [26]. Notably, the combination of letrozole (LET) and crocin (Cro) partially restored cell viability, suggesting potential synergistic effects. Although the AZO + LET + Cro combination exhibited the highest viability among treatment groups, the differences were not statistically significant, indicating the need for further research to elucidate the specific interactions and the impact of these pharmacological agents (Figure 3).

**Figure 4:**
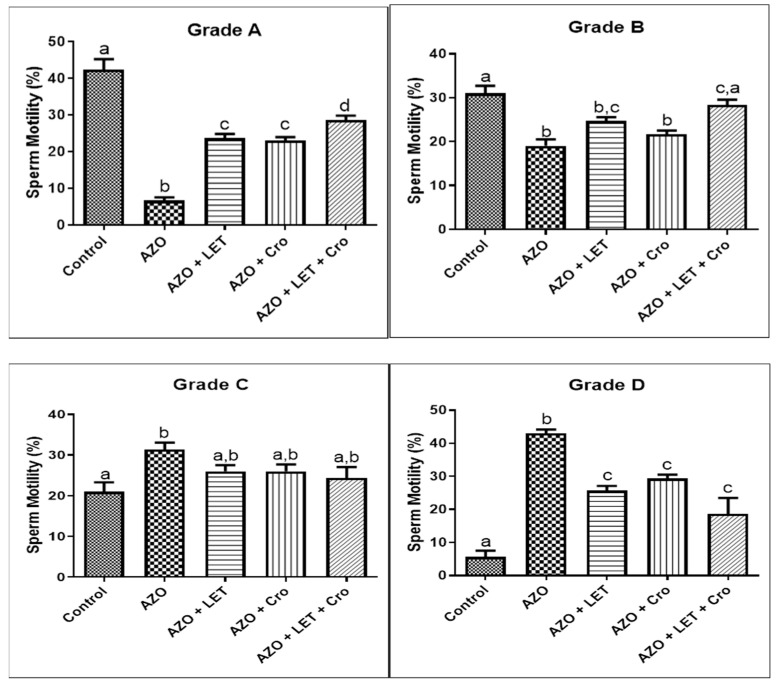
The sperm motility rate in the studied groups. Dissimilar letters denote statistical differences (*p* < 0.05). The letters (a, b, c, etc.) above the bars represent statistically significant groupings. Bars with identical letters are not significantly different, while bars with dissimilar letters have a statistically significant difference (at the *p* < 0.05 level, as typically indicated in the figure legend or Materials and Methods Section).

An analysis of sperm motility across Grades A, B, C, and D revealed distinct responses to letrozole (LET) and crocin (Cro) treatments in the azoospermia model. Significant differences were observed in Grade A (*p* < 0.0001), with the control group exhibiting the highest motility. The LET and Cro treatments yielded modest improvements. Significant differences were noted in Grade B (*p* = 0.0004), with the combination treatment (LET + Cro) achieving motility levels comparable to those of the control group. In contrast, no significant differences were noted in Grade C (*p* = 0.0576) despite unexpectedly high motility in the AZO group. In Grade D, significant differences were observed once more (*p* < 0.0001). However, the AZO group displayed higher motility compared to the other treatments, suggesting varied efficacy based on the initial motility grade (Figure 4).

These differences may reflect the inherent variability in the AZO model, indicating residual spermatogenesis or compensatory motility mechanisms in some individuals. The lower motility observed in the treatment groups relative to AZO within these grades could stem from different responses to letrozole and crocin based on the initial sperm quality. The effectiveness of treatments may vary depending on the degree of sperm damage, indicating a nuanced interaction between therapeutic agents and sperm health.

Gregoriou et al. [27] corroborate this finding, suggesting that the LET mechanism involves the stimulation of hormones critical for spermatogenesis. Interestingly, while the treatment was associated with enhanced motility in the azoospermia model, some evidence suggests that it may also impair motility under certain pathological conditions [28]. Although the LET and Cro treatments demonstrated modest improvements, the intriguing aspect of their efficacy warrants further research. Furthermore, the findings indicate significant variances in sperm viability across treatment groups, emphasizing the need for further investigation into treatment mechanisms. The improved viability observed with treatments aligns with the findings of Salahshoor et al. [29] and Mehdipour et al. [30], highlighting the potential role of Cro’s antioxidant properties in promoting healthier sperm profiles by combating oxidative damage, a crucial factor in semen quality.

**Figure 5:**
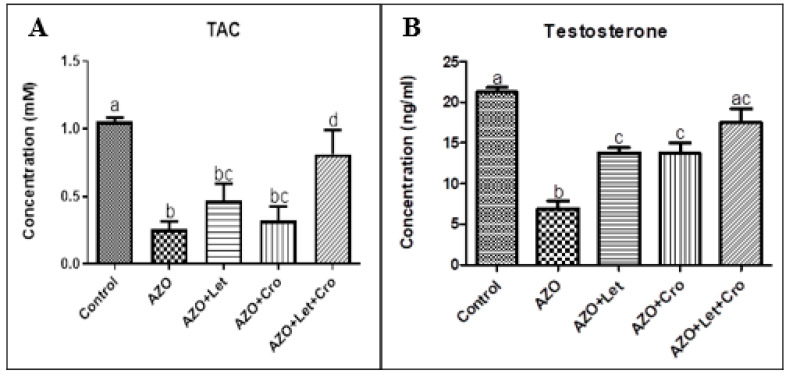
(**A**) The total antioxidant enzyme activity, (**B**) Variations in the testosterone hormone levels in different groups. Dissimilar letters denote statistical differences (*p* < 0.05). The letters (a, b, c, etc.) above the bars represent statistically significant groupings. Bars with the same letter are not significantly different, while bars with dissimilar letters have a statistically significant difference (at the *p* < 0.05 level, as typically indicated in the figure legend or Materials and Methods Section).

The combined letrozole and crocin azoospermia treatment significantly enhanced the total antioxidant enzyme activity compared to the isolated azoospermia group (*p* < 0.05) (Figure 5A), indicating a protective effect against oxidative stress [31]. In contrast, the azoospermia + letrozole group exhibited reduced antioxidant enzyme activity compared to the control group (*p* < 0.005). The significant increase in testosterone levels across treatment groups, particularly in the combined letrozole and crocin group (Figure 5B), suggests improved Leydig cell function and hormonal support for spermatogenesis [32,33]. These findings highlight the synergistic effects of letrozole and crocin in promoting antioxidant defenses and restoring hormonal balance, which are critical for mitigating oxidative stress and supporting spermatogenesis [34].

Maintaining appropriate testosterone levels and the functionality of *androgen receptors (ARs)* is essential for spermatogenesis, influencing both the growth and differentiation of spermatogenic cells. Recent investigations have elucidated that busulfan exposure can significantly impact *CYP19A1 (Cytochrome P450 Family 19 Subfamily A Member 1)* gene expression, which encodes aromatase, an enzyme responsible for the conversion of androgens to estrogens [34]. *Androgen receptor* dysregulation and *CYP19A1* expression can lead to hormonal imbalances, further compounding the adverse impact on spermatogenesis. *CYP19A1* encodes aromatase, the enzyme responsible for estrogen synthesis. Under normal physiological conditions, *CYP19A1* expression is tightly regulated. However, aberrant expression, even in the absence of other hormonal perturbations, can lead to significant estrogen imbalances. For instance, conditions such as aromatase excess syndrome and certain cancers are associated with increased *CYP19A1* activity, resulting in pathologically high estrogen levels. These imbalances can lead to precocious puberty in females, gynecomastia in males, and an increased risk of estrogen-dependent tumors. Thus, while the mechanisms are complex, increased *CYP19A1* expression alone is sufficient to disrupt hormonal homeostasis.

**Figure 6:**
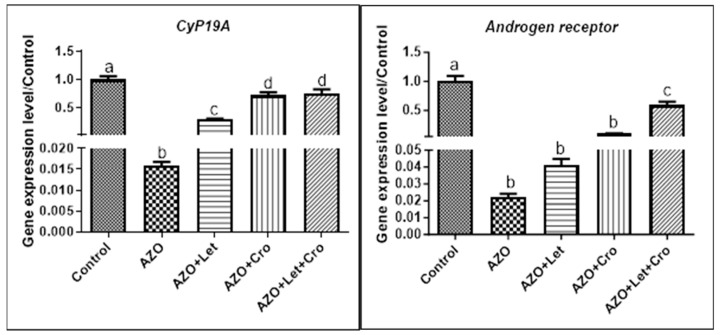
The results obtained from the expression levels of the *CYP19A1* genes and *Androgen receptor* in different groups. Dissimilar letters denote statistical differences (*p* < 0.05). The letters (a, b, c, etc.) above the bars represent statistically significant groupings. Bars with identical letters are not significantly different, while bars with dissimilar have a statistically significant difference (at the *p* < 0.05 level, as typically indicated in the figure legend or Materials and Methods Section).

The study’s findings indicate that the combination of letrozole and crocin treatment significantly increased *CYP19A1* expression compared to the azoospermia group (*p* < 0.05) and increased *androgen receptor* expression in the AZO + Let + Cro group relative to the other groups (*p* < 0.001). In the control group, *CYP19A1* expression was significantly higher (*p* < 0.001). The expression of androgen receptors in the control group was also significantly higher compared to the other groups (*p* < 0.001) (Figure 6). Furthermore, studies have indicated that busulfan treatment is associated with a marked decrease in *androgen receptor* expression in testicular tissues, which could diminish the responsiveness of Sertoli cells and Leydig cells to testosterone, leading to impaired support for spermatocytes, ultimately reducing sperm production. The consequences of diminished *androgen receptor* signaling may include reduced cell proliferation, disrupted communication among testicular somatic and germ cells, and hindered differentiation processes. Additionally, reduced *androgen receptor* activity may adversely impact the transcription of genes vital for testicular functionality, exacerbating the detrimental impact of busulfan on spermatogenesis [35].

The interplay between *androgen receptor* signaling and *CYP19A1* gene expression is being increasingly recognized as a critical factor. Evidence suggests that the *androgen receptor* directly influences the transcriptional regulation of *CYP19A1*, thereby modulating estrogen levels in the testes [36]. The perturbations induced by busulfan may lead to an imbalanced estrogen-to-testosterone ratio, further compromising spermatogenesis [37]. Elevated estrogen levels, resulting from increased androgen aromatization, can have various effects on the hypothalamic–pituitary–gonadal (HPG) axis, potentially inhibiting gonadotropin release and, by extension, testosterone synthesis [38].

The intricate regulatory mechanisms involving androgen receptors and *CYP19A1* gene expression play a pivotal role in the pathophysiology of busulfan-induced azoospermia. Understanding these molecular interactions is integral for developing targeted therapeutic strategies aimed at mitigating the adverse impact of chemotherapeutic agents on male reproductive health [39]. Further research is necessary to elucidate optimal strategies for restoring testosterone-mediated effects within the testes and assessing potential avenues to reverse the detrimental impact of busulfan, ultimately advancing treatment options for affected individuals.

## 4. Conclusions

The findings of this study indicate that the control rat sample exhibited typical Leydig cell morphology and function. However, the azoospermia group displayed a reduction in Leydig cell populations, accompanied with disruptions in cytoplasmic structure and the basement membrane. These changes may adversely impact the testicular microenvironment and the integrity of germ cells. While the letrozole (LET) and crocin (Cro) treatments partially reduced the effects of azoospermia, they were associated with an increase in testicular fibrosis in the AZO and AZO + Cro groups, showing approximately 37% and 34% fibrosis, respectively, compared to around 5% in the control group. Notably, the combined LET and Cro treatment resulted in a decrease in fibrosis to approximately 15%.

Furthermore, the combined treatment was associated with increased antioxidant enzyme activity and enhanced expressions of the *CYP19A1* gene and androgen receptors, potentially indicating protective effects against oxidative stress. Although the treatment demonstrated a trend toward improved sperm motility, these changes were not statistically significant overall. Consequently, further research is warranted to fully elucidate the mechanisms of action and potential therapeutic implications of these compounds in azoospermia treatment.

## Figures and Tables

**Table 1:** List of primers for real-time PCR.

Genes	Sequence	3′-5′
r-GAPDH	F	AGGTCGGTGTGAACGGATTTG
	R	TGTAGACCATGTAGTTGAGGTCA
r-Androgen receptor	F	GAGGGCATCAGAGGGGAAAAG
	R	TCACCGAAGAGGAAAGGGC
r-CYP19A	F	GTCCATTCCAGCACCCTTACA
	R	CATGGGGTTCAGCATTTCCAA

## Data Availability

The data included in this study were within the framework of routine clinical activities. The study was carried out with the formal approval of the Institutional Review Board of our clinic following the principles of the Declaration of Helsinki.
